# *Beta vulgaris* rubra L. (Beetroot) Peel Methanol Extract Reduces Oxidative Stress and Stimulates Cell Proliferation via Increasing VEGF Expression in H_2_O_2_ Induced Oxidative Stressed Human Umbilical Vein Endothelial Cells

**DOI:** 10.3390/genes12091380

**Published:** 2021-08-31

**Authors:** Laila Naif Al-Harbi, Subash-Babu Pandurangan, Alhanouf Mohammed Al-Dossari, Ghalia Shamlan, Ahmad Mohammad Salamatullah, Ali A Alshatwi, Amna Abdullah Alotiby

**Affiliations:** 1 Department of Food Science and Nutrition, College of Food and Agricultural Sciences, King Saud University, Riyadh 11451, Saudi Arabia; sbpandurangan@ksu.edu.sa (S.-B.P.); 438203060@student.ksu.edu.sa (A.M.A.-D.); shamLana@ksu.edu.sa (G.S.); asalamh@ksu.edu.sa (A.M.S.); alshatwi@ksu.edu.sa (A.A.A.); 2 Department of Haematology and Immunology, Faculty of Medicine, Umm Alqura University, Makkah 24237, Saudi Arabia; aamogaty@uqu.edu.sa

**Keywords:** beetroot, oxidative stress, mitochondria, angiogenesis, inflammation

## Abstract

The antioxidant capacity of polyphenols and flavonoids present in dietary agents aids in arresting the development of reactive oxygen species (ROS) and protecting endothelial smooth muscle cells from oxidative stress/induced necrosis. Beetroot (*Beta vulgaris* var. rubra L.; BVr) is a commonly consumed vegetable representing a rich source of antioxidants. Beetroot peel’s bioactive compounds and their role in human umbilical vein endothelial cells (HUVECs) are still under-researched. In the present study, beetroot peel methanol extract (BPME) was prepared, and its effect on the bio-efficacy, nuclear integrity, mitochondrial membrane potential and vascular cell growth, and immunoregulation-related gene expression levels in HUVECs with induced oxidative stress were analysed. Gas chromatography–mass spectroscopy (GC-MS) results confirmed that BPME contains 5-hydroxymethylfurfural (32.6%), methyl pyruvate (15.13%), furfural (9.98%), and 2,3-dihydro-3,5-dihydroxy-6-methyl-4H-Pyran-4-one (12.4%). BPME extract effectively enhanced cell proliferation and was confirmed by MTT assay; the nuclear integrity was confirmed by propidium iodide (PI) staining assay; the mitochondrial membrane potential (Δψ_m_) was confirmed by JC-1 staining assay. Annexin V assay confirmed that BPME-treated HUVECs showed 99% viable cells, but only 39.8% viability was shown in HUVECs treated with H_2_O_2_ alone. In addition, BPME treatment of HUVECs for 48 h reduced mRNA expression of lipid peroxide (LPO) and increased NOS-3, Nrf-2, GSK-3β, GPX, endothelial nitric oxide synthase (eNOS) and vascular cell growth factor (VEGF) mRNA expression levels. We found that BPME treatment decreased proinflammatory (nuclear factor-κβ (F-κβ), tissue necrosis factor-α (TNF-α), toll-like receptor-4 (TLR-4), interleukin-1β (IL-1β)) and vascular inflammation (intracellular adhesion molecule (ICAM), vascular cell adhesion molecule (VCAM), EDN_1_, IL-1β)-related mRNA expressions. In conclusion, beetroot peel treatment effectively increased vascular smooth cell growth factors and microtubule development, whereas it decreased vascular inflammatory regulators. BPME may be beneficial for vascular smooth cell regeneration, tissue repair and anti-ageing potential.

## 1. Introduction

Angiogenesis is the physiological process of vasculogenesis from the body’s existing vasculature [1]. It is essential not only for embryonic development and reproduction but also for cell cycle and tissue repair [2,3]. However, it is associated in the pathogenesis of various diseases, such as the tumour growth, rheumatoid arthritis and various ischaemic and inflammatory diseases [3,4,5]. The vascular endothelium plays an important role in maintaining vascular haemostasis by regulating blood vessel tone and immune and inflammatory reactions [6,7]. Endothelial cells (ECs) are thin monocellular layers that line all inner surfaces of blood vessels and produce a variety of molecules that act locally or at distant sites [7]. The endothelium is fundamental for body homeostasis, and any alteration in the endothelium cell response leads to the primary events of inflammatory and vascular disease processes, such as atherosclerosis and hypertension [6,8,9]. These diseases cause oxidative stress, which alters the EC structure and function integrity and leads to endothelial dysfunction [9]. Human umbilical vein ECs (HUVECs) have been widely used as a model for the human vascular endothelium related study. Moreover, they represent a useful model for studying the main biological pathways involved in endothelium function [10]. 

Bioactive compounds and phytochemicals are abundantly found in fruit, vegetables, green herbs, and many plants, which exhibit numerous health benefits, such as anti-inflammatory, antioxidant, anticarcinogenic and angiogenic properties [11,12,13]. In this regard, red beetroot (*Beta vulgaris* var. rubra L.; BVr) belongs to Amaranthaceae family and is classified as one of the best sources of high levels of antioxidants [14,15]. Specifically, it contains multiple phytochemicals that are biologically active, including betalains, flavonoids, polyphenols, therapeutic enzymes, ascorbic acid, dehydroascorbic acid (DHAA), and inorganic nitrate (NO_3_) [16,17,18]. Moreover, it provides valuable essential nutrients, such as potassium, calcium, magnesium, sodium, iron, zinc, phosphorous, copper and manganese [19]. Several studies have reported that red beetroot extract (root) has numerous beneficial effects because of its hypoglycaemic, lipid-lowering, anti-inflammatory, antihypertensive and antiproliferative properties [20,21,22]. These beneficial properties can all be linked to the free radical-scavenging abilities of the bioactive compounds. Thus, consumption of red beetroot has been linked to numerous nutritional and health benefits. Because of its nutritional value, it may be used as a functional food source against oxidative stresses that induce chronic metabolic diseases, such as type 2 diabetes and cardiovascular disease [23]. 

Based on the literature review, *Beta vulgaris* possess potent antioxidant, immunoregulatory and angiogenic properties. Apigenin has been found in beet leaves; it has antiproliferative effects in liver and intestinal cells, and it can improve high fat diet induced obesity comorbidity via AMPK activation [24,25]. De silva et al., (2020) [26] identified that *Beta vulgaris* protects vascular ECs from externally induced oxidative stress, which may be due to the combined effect of several bioactive compounds present in this plant. Until now, the mechanistic action for EC proliferation and angiogenesis effect of *Beta vulgaris* root peel has been under-researched. Hence, we aimed to conduct the present study to examine the vascular cell proliferation, microtubule development, oxidative stress and angiogenesis capacity related to *Beta vulgaris* root peel using cellular morphology and gene expression analysis in HUVECs. The angiogenic effects of red beet root peel methanol extract associated with nuclear integrity, microtubule development, mitochondrial efficiency and cell cycle stimulation in human vascular ECs are explored. 

## 2. Materials and Methods

### 2.1. Preparation of Beetroot (Beta vulgaris rubra *L.*) Methanol Extract 

Samples of fresh beetroot (*Beta vulgaris* var. rubra L.; BVr.) were initially obtained from vegetable stores, Riyadh, Kingdom of Saudi Arabia (KSA). The fresh beetroots were washed with distilled water to eliminate the stems and contaminants. The outer skin was peeled to removed and cut into small pieces. Samples were dried in a hot air oven at 40 °C and then ground to powder using an electronic blender. Then, 500 g of the powder was extracted in a sterile bottle contained 1 L of methanol (Sigma, St. Louis, MO, USA) for 24 h at room temperature on a shaker and repeated three times. Afterwards, a Whatman filter (Whatman, Clifton, NJ, USA) was used to filter the extract. Finally, by reducing the pressure, the solvent was separated from the extract, and the extract was collected as a solid dry substance after evaporation of methanol. The extracted sample was stored in a refrigerator at 4 °C until further use. 

### 2.2. Gas Chromatography & Mass Spectroscopy Analysis 

The beetroot peel methanol extract (BPME) was injected into a silica capillary column (30 m × 0.25 mm I.D. × 0.25 μm film thickness) of GC-MS instrument (Agilent 6890N/5973I, California, CA, USA) with a mass selective detector to detect the chemical compositions. The instrument’s temperature was set as initial 70 °C, holding 2 min, to 305 °C at 20 °C/min, followed by holding for 1 min. The total GC running time was set to 45 min with the helium gas (99.999%) as a carrier gas (a constant flow rate of 1.2 mL/min), 250 °C as the injector temperature and 230 °C as an ion source temperature. Based on the GC-MS spectrum, the relative percentage of the corresponding component was calculated, and the mass spectra of the unknown component were identified by comparison with the known 62,000 patterns available in the National Institute of Standard and Technology computer library (NIST08). 

### 2.3. Cell Culture Materials and Chemicals

HUVECs were purchased from the American Type Culture Collection (ATCC, Manassas, VA, USA). Cell culture materials such as Dulbecco’s Modified Eagle Medium (DMEM), EDTA, trypsin, and others were obtained from Gibco (Paisley, UK). Penicillin–streptomycin (PS) and foetal bovine serum (FBS) were purchased from Hyclone Laboratories, USA. Chemicals used in the molecular biology experiment were obtained from Sigma-Aldrich, especially, MTT [3-(4,5-dimethylthiazol-2-yl)-2,5-diphenyltetrazolium bromide], PI and JC-1 stain. The SYBR Green PCR Master Mix and the cDNA synthesis kit were obtained from Qiagen (Hilden, Germany).

### 2.4. HUVECs

HUVECs were cultured in DMEM and supplemented with 1% PS and 10% FBS complex. Cells were incubated in a humidified atmosphere at 37 °C, 5% CO_2_, and sub-cultured approximately every 3 days. 

### 2.5. Cell Viability and Cell Proliferation by MTT Assay 

HUVECs (1 × 10^4^ cells/well) were cultured with maintenance medium and allowed to adhere overnight in 96-well culture plate. Then, the medium was replaced with new culture medium containing increasing concentrations of BPME (0, 0.05, 0.1, 0.2, 0.4, 0.8, 1.6 and 3.2 µg/mL) as per the MTT assay plate map and incubated for 24 and 48 h; untreated cells were used as controls. After the incubation period, the experimental cells were treated with 20 µL/well of 5 mg/mL of MTT (3-[4,5-dimethylthiazol-2-yl]-2,5-diphenyltetrazolium bromide that was dissolved in dimethyl sulfoxide (DMSO)) and additionally incubated for 4 h at 37 °C. Then, the medium was discarded, and the produced purple formazan was dissolved in 100 µL of 100% DMSO. The absorbance of the solution was measured using a microplate reader (Thermo Scientific, Waltham, MA, USA) at 570 nm wavelength. The percentage (%) of cell proliferation was calculated by the following equation: (absorbance of the sample/mean absorbance of the control) × 100.

### 2.6. Experimental Design

**Figure 1:**
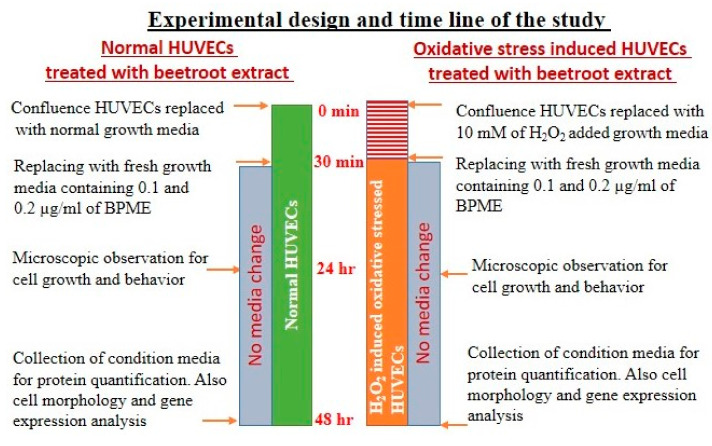
Diagrammatic representation of experimental design and timeline for the present study.

According to the present cell proliferation assay, tested lower concentration of BPME (0.1 and 0.2 µg/mL) showed proliferating HUVECs and microtubule morphology without toxicity. Volumes of 0.1 and 0.2 µg/mL dose of BPME were selected and treated with normal HUVECs and 10 mM of H_2_O_2_-induced oxidative-stressed HUVECs for 48 h to determine the cell proliferation, anti-inflammatory, angiogenic and apoptotic potentials (Figure 1). Vehicle control was also maintained for 48 h in both the groups. Quercetin (10 μM) was used as a reference control in both experimental groups. After incubation, the untreated and experimental cells were analysed for cell and nuclear morphology and mitochondrial membrane potential using a BD^TM^ MitoScreen (JC-1) Kit; apoptosis was determined by the Annexin V/apoptosis-based cell sorting method in flow cytometry. The oxidative stress, proinflammatory and angiogenesis-related gene expression levels were investigated.

### 2.7. Propidium Iodide Staining Assay for Nuclear Damage

Cellular morphologies for characteristic nuclear damage, pyknosis or apoptotic morphological changes after treatment with 0.1 and 0.2 µg/mL of BPME (with or without H_2_O_2_) in HUVECs were determined using PI staining analysis under inverted florescence microscopy, as described by Leite et al. [27]. 

### 2.8. Assay of Mitochondrial Membrane Potential (*Δ*ψ_m_) by JC-1 Dye Staining 

The potential of the mitochondrial membrane (Δψ_m_) was determined by JC-1 assay to assess mitochondrial efficiency in vehicle control and 0.1 and 0.2 µg/mL of BPME-treated HUVECs (with and without H_2_O_2_). Briefly, the JC-1 staining solution was mixed with a similar volume of culture medium and then added to the experimental HUVECs and incubated in the dark for 20 min at 37 °C. Then, the unbound JC-1 dye was gently washed two times using 200 μL of JC-1 staining wash buffer at 4 °C. Afterwards, the accumulation of j-aggregated against JC-1 staining was observed under fluorescence microscopy using a fluorescence microscope, and images were captured. In addition, the potential of the mitochondrial membrane was measured in flowcytometry using the BD^TM^ MitoScreen (JC-1) Kit.

### 2.9. Annexin V/apoptosis Analysis Using Flow Cytometry

The flow cytometry-based Annexin V/PI detection kit (Sigma Chemicals, USA) method was used to quantify viable, proapoptotic, early apoptotic and necrotic cells. Oxidative stress induced HUVECs (1 × 10^5^/well) were plated in 24-well plates and incubated with BPME (0.1 and 0.2 μg/mL) or vehicle control for 48 h. After incubation, cells were incubated in 400 μL of 5 μL Annexin V-fluorescein isothiocyanate (FITC) and 5 µL of PI containing binding buffer; following this, the cells were kept for 15 min at room temperature (RT) in the dark. The cells were analysed by flow cytometry (BD Biosciences, San Jose, CA, USA) to identify apoptotic (PI negative and Annexin V positive) and late apoptotic (PI positive and Annexin V positive) cells [28].

### 2.10. Quantitative Real-Time PCR Analysis

Fastlane^®^ Cell to cDNA kit (Qiagen, Hilden, Germany) was used to extract total RNA and synthesis cDNA from vehicle control, BPME-treated HUVECs (with and without H_2_O_2_) using a quantitative PCR (qPCR) semiautomated instrument (Applied Biosystems, Foster City, CA, USA). Expression levels of oxidative stress including (lipid peroxide, NOS-3), antioxidant (Nrf-2, GSK-3β and GPx), proinflammatory (nuclear factor-κβ (NF-κβ), tumour necrosis factor-α (TNF-α), interleukin-1β (IL-1β), vascular cell growth factor (VEGF), toll-like receptor-4 (TLR-4)), and vascular inflammation (intracellular adhesion molecule (ICAM), vascular cell adhesion molecule (VCAM), EDN_1_ and endothelial nitric oxide synthase (eNOS))-related genes and the reference gene, β-actin, were analysed in HUVECs and quantified by the method of Yuan et al. [29]. The amplification values (ΔCt) were calculated by the difference between Ct (treated) and Ct (control). Gene expression was plotted using the expression of 2^−ΔΔCt^ value.

### 2.11. Statistical Analysis 

All the experiments were triplicated, and the resulting data were expressed as mean values ± standard deviation (SD). The statistical analysis of differences among the groups was performed by one-way analysis of variance (ANOVA) using SPSS software (Version 28.5, SAS Institute Inc., Cary, NC, USA). Then, Tukey’s multiple comparison test was conducted if significant differences were found. All the results were presented as the mean ± SD for six replications in each group. A *p* value < 0.05 was considered significant [30]. 

## 3. Results

### 3.1. Bioactive Molecules in BPME

**Figure 2:**
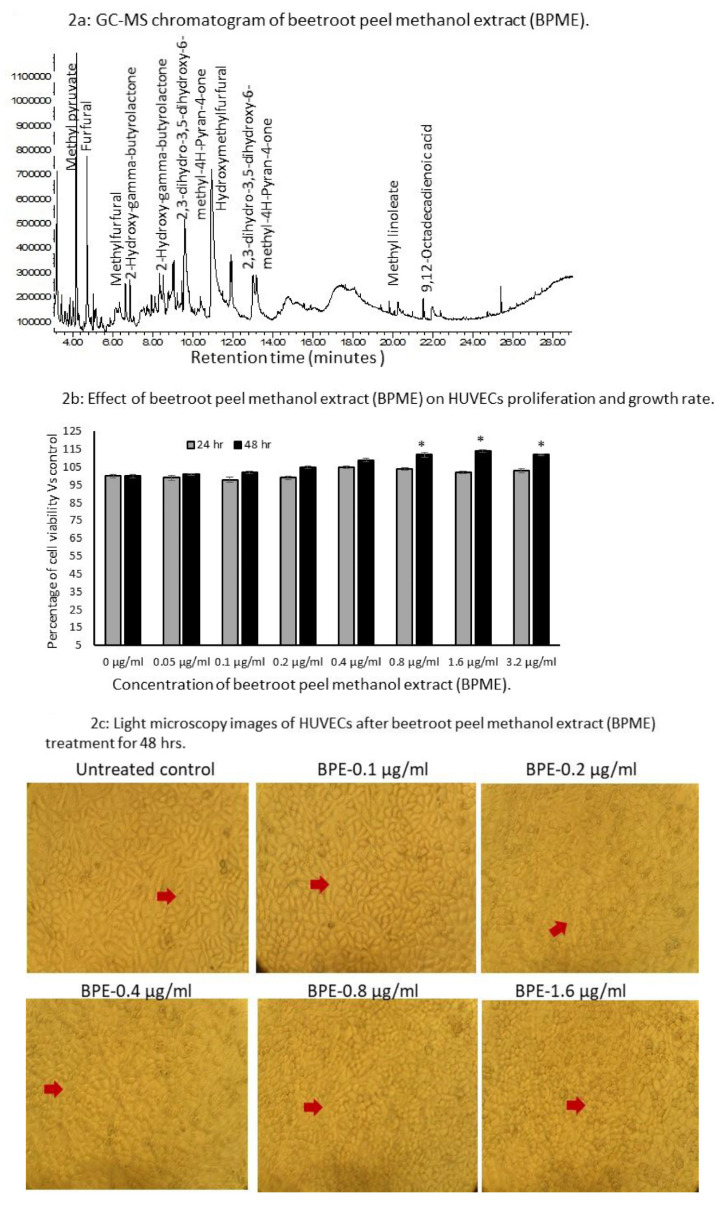
Results for GC-MS chromatogram of beetroot peel methanol extract (**a**), in vitro cell proliferation (**b**) and light microscopy images for cell morphology (**c**) in HUVECs treated with increasing concentration of beetroot peel methanol extract (BPME) (uniform shape of adherent and proliferating cells mentioned by red arrowhead). All values are means ± SD (*n* = 6). * *p* ≤ 0.05 by comparison with vehicle control.

The chemical constituents of BPME were confirmed using GC-MS (Turbomass, PerkinElmer). The chemical composition of beetroot peel extract was determined by comparing the mass spectra available with the National Institute of Standard and Technology (NIST) Spectral database. The GC-MS results confirmed that the BPME contained hydroxyacetone (8.18%), 5-hydroxymethylfurfural (32.6%), methyl pyruvate (15.13%), beta-d-allopyranose (1.48%), furfural (9.98%), 2-hydroxy-gamma-butyrolactone (1.32%), and 2,3-dihydro-3,5-dihydroxy-6-methyl-4H-Pyran-4-one (12.4%; Figure 2a, Table 1).

### 3.2. Cell Proliferation

The in vitro cell proliferation potential of BPME against HUVECs is presented in Figure 2b. No significant cell growth inhibition was observed in the experimental groups compared with the vehicle control. It was confirmed by the present study that increasing the concentration of BPME treated with HUVECs resulted in increased cell proliferation and viability after 48 h (112%) when compared with 24 h (103%) of treatment. In addition, the light microscopic images of BPME-treated HUVECs after 48 h confirmed the normal cells with uniform shape of adherent cell morphology, increased number of proliferating (replication) cells without any damaged were apparent (Figure 2c).

### 3.3. Analysis of Cell and Nuclear Morphology, Microtubule Formation and JC-1 Staining in HUVECs 

**Figure 3:**
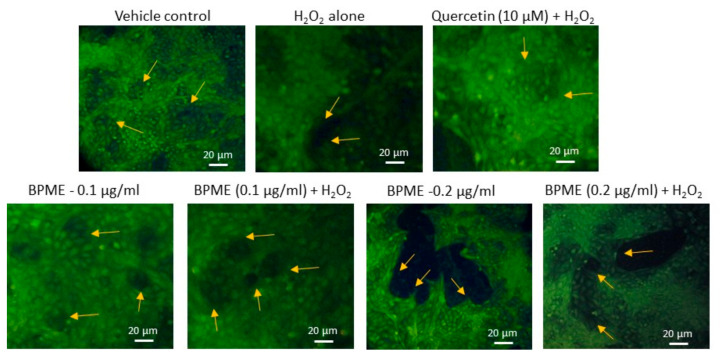
Analysis of microtube formation based on florescence microscopic in vehicle control, 0.1 and 0.2 μg/mL dose of beetroot peel methanol extract (BPME) treated normal and H_2_O_2_-induced oxidative-stressed HUVECs after 48 h. After 48 h, H_2_O_2_-induced oxidative-stressed HUVECs showed altered morphology when compared to vehicle control. Volumes of 0.1 and 0.2 μg/mL of beetroot peel methanol extract (BPME)-treated HUVECs showed normal morphology with vascular microtubules with proliferating cells resembling the common morphology of smooth muscle cell behaviour.

Figure 3 shows the morphology of microtubule development in fluorescence microscopic images. H_2_O_2_-induced oxidative-stressed HUVECs showed poor proliferation and irregular morphology of adherent cells compared with control HUVECs. Normal HUVECs treated with 0.2 µg/mL of BPME showed proliferating cells via replication or neogenesis with microtubule morphology. Meanwhile, 0.1 µg/mL dose of BPME treated cells showed 100% adherent cells with early stages of microtubules. Oxidative-stressed HUVECs treated with 0.2 µg/mL of BPME identified with proliferating new cells with microtubule morphology and decreased oxidative cellular damage. In addition, 0.1 µg/mL of BPME also increased the normal vascular cell morphology with proliferating cells.

**Figure 4:**
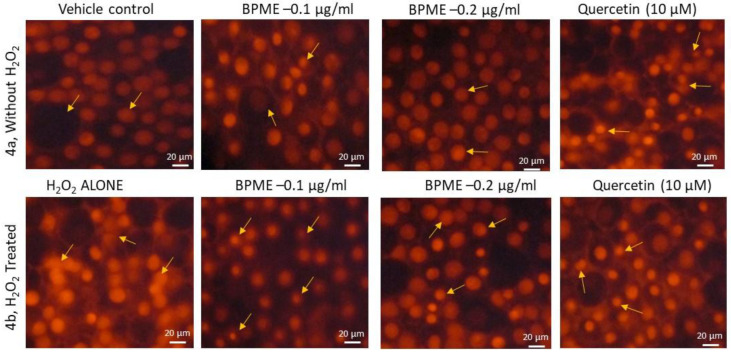
Propidium iodide (PI) staining analysis for nuclear morphology in vehicle control, 0.1 and 0.2 μg/mL of beetroot peel methanol extract (BPME)-treated normal (3a) and H_2_O_2_-induced oxidative-stressed (3b) HUVECs after 48 h. In PI staining, vehicle control showing the nucleus appeared to be normal with no signs of shrunken, pyknosis or apoptotic nucleus. In H_2_O_2_ alone, treated cells showed polarized nuclear membrane after 48 h. Treatment with 0.2 μg/mL of BPME normalized the H_2_O_2_-induced nuclear membrane polarization when compared to 0.1 μg/mL BPME or Quercetin 10 μM.

Figure 4a shows the normal morphology of the nuclear structure, with a spherical shape, in the control and BPME (0.1 or 0.2 µg/mL)-treated HUVECs. Figure 4b shows the images for PI staining of normal and HUVECs with oxidative stress induced by H_2_O_2_. H_2_O_2_-treated HUVECs show nuclei with irregular shapes, exhibiting condensation and pyknosis after 30 min. However, 0.2 µg/mL of BPME treatment for HUVECs under oxidative stress showed circular nuclei with a normal morphology. Compared with 0.2 µg/mL of BPME extract, 0.1 µg/mL of BPME had a lesser protective effect against H_2_O_2_-induced oxidative stress in HUVECs. 

**Figure 5:**
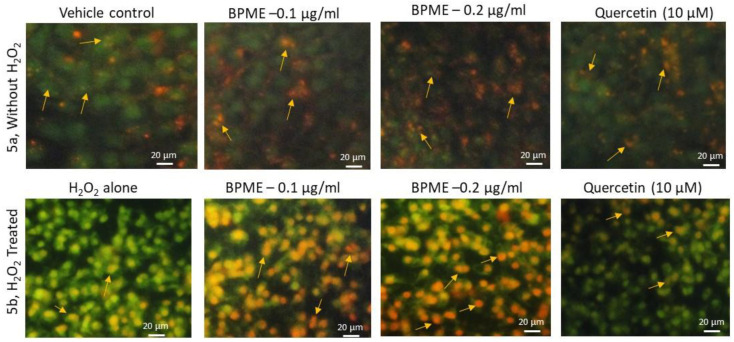
Analysis of mitochondrial membrane potential using JC-1 staining for vehicle control, 0.1 and 0.2 μg/mL of beetroot peel methanol extract (BPME) treated normal (**a**) and H_2_O_2_-induced oxidative-stressed (**b**) HUVECs after 48 h. JC-1 fluorescence images showing merged images of the red and green signals of the dye, corresponding to JC-1 in J-aggregates vs. monomeric form. We found less J-aggregates H_2_O_2_ alone treated HUVECs. In 0.2 μg/mL BPME-treated HUVECs showing high j-aggregates directly representing (high MMP, Δψ_m_) high mitochondrial membrane potential compared to 0.1 μg/mL of BPME or 10 μM of Quercetin.

Figure 5a shows the JC-1 staining results for HUVECs, including control and BPME-treated cells; the figure illustrates healthy cells with active mitochondria, as confirmed by the negatively charged mitochondria uptake of the extra mitochondrial lipophilic cationic JC-1 (green colour) and J-aggregates converted with red colour intramitochondrially. Figure 5b shows the JC-1 staining results for 0.2 µg/mL of BPME administered to HUVECs with oxidative stress induced by H_2_O_2_; the results confirmed almost 94% of negatively charged mitochondria converted the lipophilic cationic JC-1 (green colour) to red colour J-aggregates when compared with 0.1 µg/mL of BPME (61.4%) treatment or H_2_O_2_-induced oxidative-stress in HUVECs (2%). The mitochondrial membrane potential (MMP) was observed to be higher in BPME-treated cells compared with the reference drug quercetin. 

### 3.4. FACS-Assisted Mitochondrial Membrane Potential (*Δ*ψm; BD MitoScan) and Annexin V/apoptosis Analysis in HUVECs

**Figure 6:**
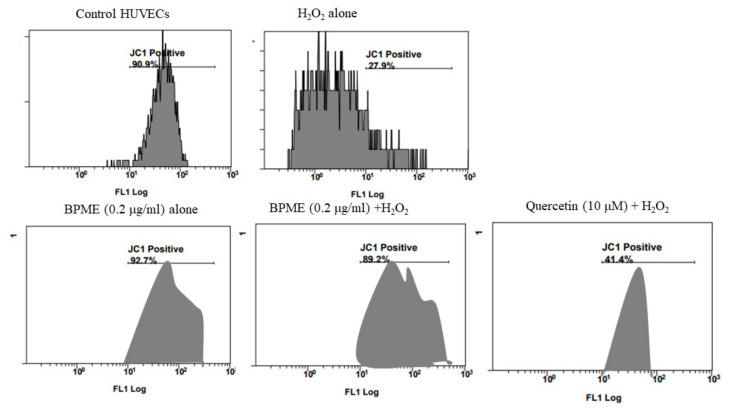
Flow cytometry-based analysis of mitochondrial membrane capacity using JC-1 (Δψ_m_, BD Mito Scan) in normal and H_2_O_2_-induced oxidative-stressed HUVECs treated with 0.2 μg/mL of BPME. Untreated cells were considered a negative control, whereas H_2_O_2_ (10 mM) was added for positive control.

Figure 6 shows the mitochondrial membrane potential capacity in BD MitoScan analysis after 0.2 µg/mL of BPME treatment of normal HUVECs and HUVECs with oxidative stress induced by H_2_O_2_. We found that 0.2 µg/mL of BPME treatment increased the MMP (Δψm) to 92.7% ± 3.7% when compared with HUVECs treated with H_2_O_2_ alone (27.9% ± 7.2%). In contrast, quercetin-treated cells showed a 41.4% ± 1.6% percentage of increased MMP (Δψm) when compared with HUVECs treated with BPME and H_2_O_2_ or H_2_O_2_ alone. 

**Figure 7:**
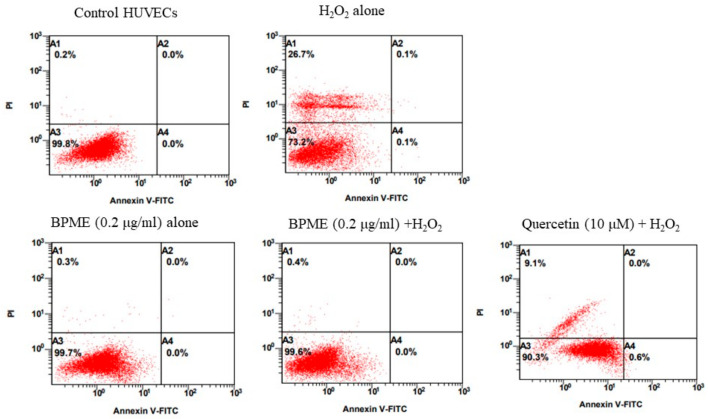
Annexin-V/propidium iodide double staining-based apoptosis, necrosis or late apoptotic characteristic analysis of BPME (0.2 μg/mL)-treated normal and H_2_O_2_-induced oxidative-stressed HUVECs. Untreated cells were considered a negative control, whereas H_2_O_2_ (10 mM) was added for positive control. (Annexin-V/apoptosis quadrants: A3: viable cells; A4: early apoptosis; A2: late apoptosis; A1: necrosis).

As shown in Figure 7, the quadrants representing the cells with Annexin V assay are as follows: quadrant A3, viable cells; A2, late apoptosis; A4, early apoptosis; and A1, necrosis. Control cells showed 99.8% ± 4.2% of viable cells, but HUVECs treated with H_2_O_2_ alone showed 73.2% ± 1.7% viable cells (A3) and 26.7% ± 3.8% necrotic cells (A1). After treatment with 0.2 µg/mL of BPME in normal HUVECs, viable cells represented 99.7% ± 3.1% of cells. A volume of 0.2 µg/mL of BPME administered to HUVECs with oxidative stress induced by H_2_O_2_ showed 99.6% ± 4.7% viable cells in quadrant A3 and 0.4% ± 0.02% necrosis in quadrant A1. Meanwhile, quercetin-treated HUVECs showed 90.3% ± 3.5% viable cells, 0.6% ± 0.02% early apoptotic cells and 9.1% ± 1.2% necrotic cells. The observed results indicated that BPME reduced the necrotic cells to 26.4% when compared with H_2_O_2_-treated HUVECs. 

### 3.5. Quantification of Gene Expression Levels in HUVECs

**Figure 8:**
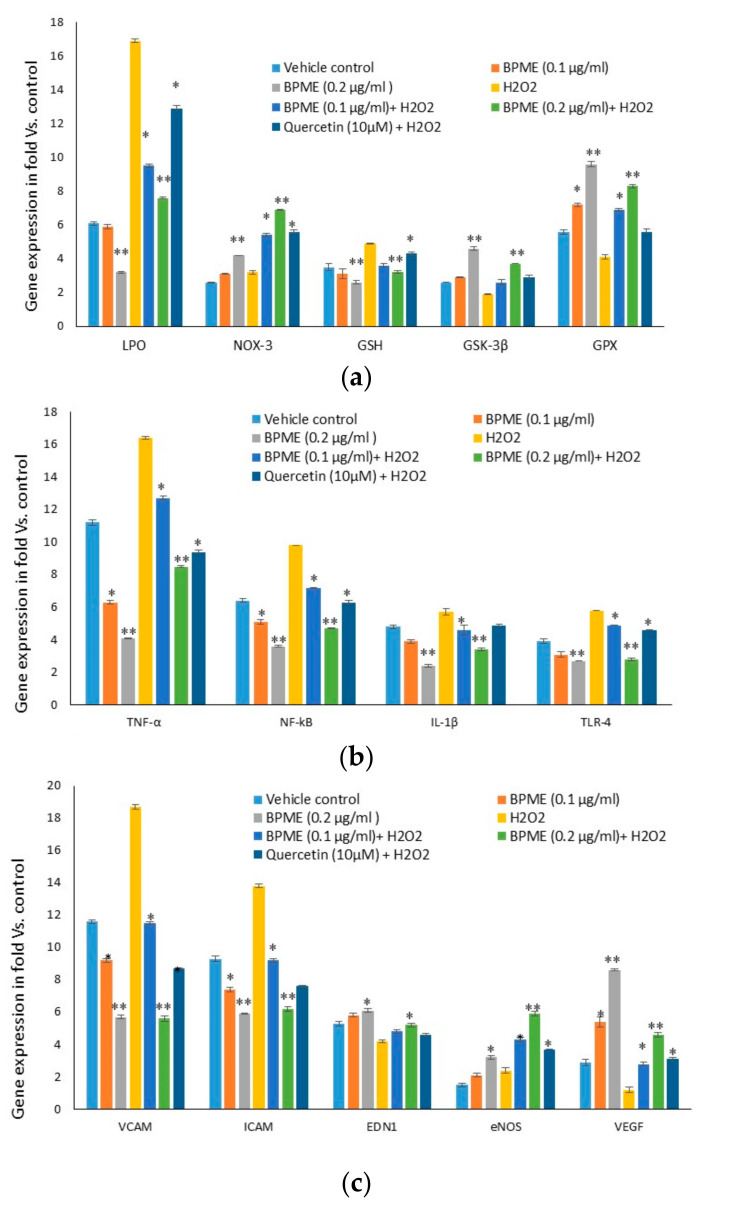
Oxidative stress, antioxidant (**a**), pro-inflammation (**b**) and vascular cell inflammation (**c**)-related gene expression levels of vehicle control, 0.1 and 0.2 μg/mL of beetroot peel methanol extract (BPME)-treated normal and H_2_O_2_-induced oxidative-stressed HUVECs after 48 h. All values are means ± SD (*n* = 6). * *p* ≤ 0.05 and ** *p* ≤ 0.001 were compared with vehicle control or H_2_O_2_ alone treated HUVECs.

Oxidative stress (LPO 3), antioxidant (NOS-3, Nrf-2, GSK-3β and GPX), proinflammatory (IL-1β, TNF-α, NF-κB and TLR-4) and vascular inflammation (VCAM, ICAM, EDN_1_, eNOS)-related and VEGF mRNA expression levels were quantified in vehicle control, 0.1 and 0.2 µg/mL of BPME and quercetin (10 μM)-treated HUVECs after 48 h (Figure 8). We found significantly (*p* ≤ 0.001) increased levels of LPO, NOS-3, NF-κB, IL-1β, TNF-α, VCAM, ICAM, EDN_1_ and eNOS expression and decreased Nrf-2, GSK-3β and GPX levels in H_2_O_2_-induced HUVECs. Treatment with 0.2 µg/mL of BPME significantly decreased oxidative stress and vascular inflammation and increased antioxidant factor-related mRNA expression when compared with oxidative-stressed HUVECs. VEGF expression levels showed a significant two-fold increase in 0.2 µg/mL of BPME-treated cells only when compared to 0.1 µg/mL of BPME. VEGF expression was not detected in HUVECs with oxidative stress induced by H_2_O_2_. The observed effect was significantly higher than that of 0.1 µg/mL BPME or quercetin (10 μM). 

## 4. Discussion

Upon extracellular or intracellular stress, reactive oxygen species (ROS) bioavailability overtakes the antioxidant defence, and oxidative stress disrupts redox signalling and control [31]. The development of oxidative stress is associated with the pathogeneses of chronic disorders, such as neurodegenerative diseases, diabetes and atherosclerosis. Such as, oxidative stress initiates endothelial dysfunction and promotes systemic inflammation and recruitment of macrophages [32]. Activated immune cells migrate into the vasculature and release cytokines and chemokines associated with vasoconstriction and remodelling of smooth muscle cell blood vessels and inflammation affecting vascular smooth muscle cells and the vascular wall [33]. Increased vascular oxidative stress ends with vascular damage, smooth muscle cell stiffness and structural elastin abnormalities. In addition, vascular oxidative stress has been stimulated in other pathological conditions, such as visceral obesity or atherosclerosis, because of increased NADPH oxidase (NOX-2) activity in perivascular adipose tissue [34]. Vascular oxidative stress causes principal epigenetic changes that occur during ageing, and it ends with an early ageing process [35].

Development of ROS production and oxidative stress in the biological system largely depends on mitochondrial dysfunction, in addition to NOX-2, endothelial xanthine oxidase, uncoupled eNOS and lipoxygenase [36]. Antioxidant properties of dietary agents may neutralise ROS generation via increased antioxidant capacity [26]. *Ginko biloba* extract protects against the development of atherosclerosis by reducing ROS generation and lipoxygenase activity in OxiLDL-induced endothelial dysfunction [37]. In addition, various phenolic compounds and flavanoids from edible plants and grains have the property to scavenge ROS and lipid peroxidation [38]. Beetroot peel (*Beta vulgaris*) methanolic extract has antioxidant potential due to the availability of high fibre, anthocyanins, and flavanoids such as vitexin and betanin [39]. In the present study, BPME was selected to identify the mitochondrial-dependent mechanistic approach to discover its effect on the mitochondrial membrane potential, LPO quenching and inhibition of vascular inflammation-related mRNA expression levels.

MTT assay confirmed that BPME significantly increased the cell proliferation, as confirmed by the increased nuclear integrity in PI staining with the effective dose of 0.2 μg/mL of BPME versus tested 0.1 μg/mL of BPME. Identification of effective dose with low concentration and highest activity may be considered as physiologically safe. We found fluorescence microscopic staining of JC-1, and the mitochondrial membrane potential was restored both in normal and H_2_O_2_-induced externally stimulated oxidative-stressed HUVECs after 0.2 μg/mL of BPME treatment. Mitochondrial dysfunction alters oxidative phosphorylation, which fails to transform oxygen (O_2_·^−^) radicals to H_2_O_2_ and H_2_O by glutathione peroxidase. Because of insufficient ROS detoxification or uncontrolled ROS production, increased mitochondrial oxidative stress is linked with atherosclerosis [40]. Beetroot peel extract effectively restored the mitochondrial membrane potential, which successfully increased ROS detoxification and H_2_O_2_ generation. 

Annexin V/PI staining analysis confirmed that BPME treatment maintained the viable cell percentage and enhanced the cell proliferation stage both in normal HUVECs and HUVECs with oxidative stress induced by H_2_O_2_. In this context, Choo et al. [41] confirmed that after the generation of excessive ROS or exogenous H_2_O_2_ at the ischaemic site, transplanted mesenchymal stem cells (MSCs) may impair self-proliferation and multi-lineage capacity. In regenerative medicine, vascular smooth muscle cells are the major regulators of the contractile tone arteries via maintaining arterial peripheral resistance, blood pressure regulators, blood flow and the repair of arteries [42]. In addition, age-induced phenotype modulation of ECs is associated with decreased cellular contractility and increased cell senescence. Upon continuous stress or because of reduced mechanosensitivity, decreased adaptation of microenvironment signals is identified in aged smooth muscle cells [43]. The present results confirmed that the BPME treatment maintained the viable cell population, as evidenced by the angiogenesis capacity. 

The identified proliferation capacity of BPME on HUVECs has been supported by the reduction in LPO expression and increased antioxidant gene expressions. ROS and LPO are initially generated from the mitochondrial complex (I and III) and NOX-4 during cell proliferation or differentiation [44]. Excessive ROS react and damage the biomolecules, especially altering the integrity of genomic DNA, which is critical for cellular proliferation and functions [45]. However, it has been confirmed that dietary intake of antioxidant polyphenols, such as epigallocatechin and tocopherol, protect the cells from oxidative stress and increase proliferation capacity [46]. In our study, the mRNA expression levels of LPO decreased, and NOS-3, Nrf-2 and eNOS were found to be increased two-fold in HUVECs with oxidative stress induced by H_2_O_2_. eNOS is the predominant isoform of NOS, responsible for most of the NO·products in smooth muscle cells and vascular tissues. NO· dilates all types of vascular vessels and protects platelet aggregation and leukocyte adhesion in ECs [47]. Thus far, there have been many conflicting reports about cardiovascular risk factors, and endothelial dysfunction has been associated with decreased or increased eNOS expression [48]. Elevated expression of eNOS observed upon vascular disease, which is likely to be a consequence of the excess production of H_2_O_2_. O_2_·^−^, a dismutation product, may increase eNOS expression through transcriptional and post-transcriptional mechanisms [49]. Pathogenesis of vascular disease is accompanied by an accelerated degradation of NO· after reaction with O_2_·^−^, and finally, ONOO^−^ form, leading to eNOS uncoupling and NOX enzyme dysfunction [50]. Oxidative stress has been suppressed by antioxidant enzymes, and mRNA levels of GSK-3β and GPX have been increased after BPME treatment. BPME contains multiple phytochemicals that are biologically active, including betalains, flavonoids, polyphenols, therapeutic enzymes, ascorbic acid, dehydroascorbic acid (DHAA) and inorganic nitrate (NO_3_), and these may be involved in the upregulation of antioxidant capacity in HUVECs. In this context, Cha et al. (2014) [51] reported that chlorogenic acid effectively protects against oxidative stress-induced DNA damage in human keratinocytes.

Endothelin-1 (Edn-1), an endothelium-derived vasoconstrictor, performs smooth muscle cell migration and acts as an antiapoptotic factor in cells with nitric oxide-induced stress [52,53]. The processes of vascular remodelling, migration, proliferation and extracellular matrix accumulation have been stimulated by both Edn-1 and NO [54,55]. We observed increased Edn-1 expression after BPME treatment in HUVECs with oxidative stress. Upon oxidative stress or LPO accumulation, the early stage of vascular inflammation is the adhesion of leukocytes to endothelial smooth muscle cells, it is prominent for the critical events of ischaemia and atherosclerosis [56]. It is mediated by VCAM and ICAM expressions; it has been stimulated by many of the chemokines and chemotaxis agents, such as NF-κB, IL-1β and TNF-α expressions [57]. Inhibition of IL-1β activation followed by adhesion molecule expression has been achieved by the dietary phenolic compound ellagic acid [58]. Treatment with BPME to externally stimulated HUVECs with oxidative stress significantly decreased the vascular cell-specific proinflammatory factors, such as VCAM, ICAM, NF-κB, IL-1β and TNF-α expression levels. In this context, Crespo et al. [59] reported that kaempferol and quercetin inhibited pro-inflammatory genes, such as VCAM, ICAM, NF- κB and IL-1β expressions, respectively. Overall, the inhibition of oxidative stress and vascular inflammation related gene expression potential of BPME favoured the expression of vascular cell growth factors and potentially aided vascular cell proliferation and growth. 

## 5. Conclusions

The present findings confirm that the increased expression of antioxidant genes was associated with quenching of oxidative stress, aiding to overcome the impairment of HUVEC proliferation and angiogenesis. Black garlic containing hydroxymethylfurfural supresses the inflammatory effect of TNFα-induced monocyte cell adhesion to HUVECs and further suppresses ROS generation, VCAM-1 expression and NF-κB activation [60]. In addition, He et al. [61] confirmed that hydroxymethylfurfural has the potential to protect ECs from hypoxia. Beetroot peel has also been identified to comprise flavonoids, furan, and antioxidant components, such as 5-hydroxymethylfurfural, methyl pyruvate, furfural and 2,3-dihydro-3,5-dihydroxy-6-methyl-4H-Pyran-4-one; these components are responsible for the enhanced antioxidant capacity and suppression of proinflammatory vascular smooth muscle cell adhesion molecules. Beetroot peel has been used as a stimulant for antioxidant pools to quench the external stimulus or internal pathological stimulus of peroxidative cellular stress. Our findings evidenced that the components of beetroot aids in reducing metabolic stress and inflammation in HUVECs, which may be beneficial for vascular cell proliferation and angiogenesis. 

## Figures and Tables

**Table 1:** GC-MS analysis of beetroot peel methanol extract.

Compound Name	Chemical Formula	Molecular Weight, g/mole	RT (min)	%Area
Hydroxyacetone	C_3_H_6_O_2_	74.08	3.221	8.18
METHYL ISOBUTYRATE	C_5_H_10_O_2_	102.13	3.634	1.09
Triethylene glycol	C_6_H_14_O_4_	150.17	3.742	0.61
4-hydroxy-2-Butanone	C_4_H_8_O_2_	88.11	4.035	2.02
Methyl pyruvate	C_4_H_6_O_3_	102.09	4.2	15.13
2-(2-Aminoethoxy)ethanol	C_4_H_11_NO_2_	105.14	4.582	0.45
Furfural	C_5_H_4_O_2_	96.08	4.735	9.98
Dodecyl 2-methoxyethyl phthalate	C_23_H_36_O_5_	392.5	4.945	0.50
4-Ethoxy-2-methylamino-2,4,6-cycloheptatrienone	C_10_H_13_NO_2_	179.219	5.059	0.88
1,6;2,3-Dianhydro-4-O-acetyl-.beta.-d-allopyranose	C_8_H_10_O_5_	186	5.174	1.48
2-Cyclopentene-1,4-dione	C_5_H_4_O_2_	96.08	5.46	0.67
Butanoic acid, 3-hydroxy-3-methyl-	C_5_H_10_O_3_	118.13	5.74	0.42
Butyrolactone	C_4_H_6_O_2_	86.09	5.912	0.59
2-Methylcyclopentanone	C_6_H_10_O	98.14	6.147	0.88
5-METHYLFURFURAL	C_6_H_6_O_2_	110.11	6.662	2.34
2,4-Dihydroxy-2,5-dimethyl-3(2H)-furan-3-one	C_6_H_8_O_4_	144.12	6.879	2.09
2-Hydroxy-gamma-butyrolactone	C_4_H_6_O_3_	102.09	7.394	1.32
Furaneol	C_6_H_8_O_3_	128.13	8.342	1.64
2-Ethyl-p-xylene	C_10_H_14_	134.22	8.539	0.72
1,3-Dimethyl-3,4,5,6-tetrahydro-2(1H)-pyrimidinone	C_6_H_12_N_2_O	128.17	8.59	0.34
N-Nitroso-N-methylurea	C_2_H_5_N_3_O_2_	103.08	8.794	0.92
2,3-dihydro-3,5-dihydroxy-6-methyl-4H-Pyran-4-one	C_6_H_8_O_4_	144.12	9.64	12.46
5-Hydroxymethylfurfural	C_6_H_6_O_3_	126.11	11.02	32.67
Methyl linoleate	_19_H_34_O_2_	294.5	21.524	0.71
9,12-Octadecadienoic acid	C_18_H_32_O_2_	280.4	21.982	1.50

## Data Availability

The data presented in this study are available on request from the corresponding author.
